# Alignment of Skeletal Muscle Cells Facilitates Acetylcholine Receptor Clustering and Neuromuscular Junction Formation with Co-Cultured Human iPSC-Derived Motor Neurons

**DOI:** 10.3390/cells11233760

**Published:** 2022-11-24

**Authors:** Kazunori Shimizu, Haruo Kassai, Yuhei Kamei, Kazuki Yamamoto, Takunori Nagashima, Tadayoshi Maekawa, Hirokazu Akiyama, Hiroyuki Honda

**Affiliations:** Department of Biomolecular Engineering, Graduate School of Engineering, Nagoya University, Nagoya 464-8603, Japan

**Keywords:** skeletal muscle, motor neuron, co-culture, micropatterns

## Abstract

In vitro neuromuscular junction (NMJ) models are powerful tools for studying neuromuscular disorders. Although linearly patterned culture surfaces have been reported to be useful for the formation of in vitro NMJ models using mouse motor neuron (MNs) and skeletal muscle (SkM) myotubes, it is unclear how the linearly patterned culture surface increases acetylcholine receptor (AChR) clustering, one of the steps in the process of NMJ formation, and whether this increases the in vitro NMJ formation efficiency of co-cultured human MNs and SkM myotubes. In this study, we investigated the effects of a linearly patterned culture surface on AChR clustering in myotubes and examined the possible mechanism of the increase in AChR clustering using gene expression analysis, as well as the effects of the patterned surface on the efficiency of NMJ formation between co-cultured human SkM myotubes and human iPSC-derived MNs. Our results suggest that better differentiation of myotubes on the patterned surface, compared to the flat surface, induced gene expression of integrin α7 and AChR ε-subunit, thereby increasing AChR clustering. Furthermore, we found that the number of NMJs between human SkM cells and MNs increased upon co-culture on the linearly patterned surface, suggesting the usefulness of the patterned surface for creating in vitro human NMJ models.

## 1. Introduction

The neuromuscular junction (NMJ) is a synapse formed between motor neurons (MNs) and skeletal muscle (SkM) fibers. Contraction of SkM fibers is induced by the binding of acetylcholine (ACh), which is released from the presynaptic MN, to the ACh receptors (AChRs) present on the postsynaptic SkM membrane of the NMJ. AChRs must be densely clustered in the membrane to achieve efficient neuromuscular transmission [1]. Neuromuscular disorders, including MN, neuropathies, NMJ disorders, and myopathies, are caused by MNs, NMJs, and/or SkM cell dysfunction. However, the detailed pathogenic mechanisms of various neuromuscular disorders have not been clarified and few effective treatments have been developed.

In vitro NMJ models are powerful tools for the study of various neuromuscular disorders. Conventionally, the co-culture of MNs and SkM myotubes in the same normal cell culture dish or plate is employed to form NMJ in vitro [2,3,4]. Although this method is simple and easy to perform, the low efficiency of NMJ formation between MNs and SkM myotubes hampers effective functional analysis of NMJs. Therefore, it is necessary to develop a method for increasing the efficiency of NMJ formation.

The topography of the cell culture surface controls cellular orientation and function in vitro [5,6]. The orientation of SkM myoblasts and myotubes are aligned by culturing on surfaces with micro- or nanometer-scale linear patterns [7,8,9] and mimicking the SkM tissue comprising long-aligned SkM fibers. In a pioneering paper by Ko et al. [10], the positive effects of the linearly patterned culture surface on the formation of NMJ between mouse MNs and SkM myotubes were reported. Mouse C2C12 or mouse primary myotubes and mouse neurons differentiated from mouse brain cortex-derived neural stem cells were co-cultured on Matrigel-coated poly(urethane acrylate) (PUA) substrates with a linear groove, and the positive area for anti-AChR staining on the co-cultured myotubes and the area where the neurons and AChR were colocalized increased on the grooved PUA compared to the flat PUA. However, it is unclear how a linearly patterned culture surface increases AChR clustering, one of the steps in the process of NMJ formation, and whether this increases the in vitro NMJ formation efficiency of co-cultured human MNs and SkM myotubes.

In this study, we investigated the effects of a linearly patterned culture surface on AChR clustering in myotubes and examined the possible mechanism underlying the increase in AChR clustering using gene expression analysis. Furthermore, we investigated the effects of linearly patterned culture surfaces on the efficiency of NMJ formation in co-cultured human SkM myotubes and iPSC-derived MNs.

## 2. Materials and Methods

### 2.1. Fabrication of Cell Culture Substrates with Linear Patterns

A micropatterned cell culture substrate comprising polydimethylsiloxane (PDMS) (Sylgard184; Dow Corning Corp., Midland, MI, USA) was fabricated using soft lithography, as previously reported, with some modifications [11]. We designed 5-, 10-, 15-, and 20 µm lines and spaces with a groove pattern of 1 µm depth. To create a micropatterned mold, a silicon wafer was coated with a photoresist; a 1:2 mixture of SU-8 3005 (MicroChem Corp., Newton, MA, USA) and isopropyl alcohol was spin-coated at 7000 rpm for 30 s. After heating the coated wafer for 3 min at 95 °C, it was irradiated with UV light through a photomask and subsequently heated at 65 °C for 1 min and 95 °C for 1 min. It was then soaked with an SU-8 developer to remove non-polymerized SU-8, followed by washing with isopropyl alcohol to complete the micro-patterned mold. Finally, to create a micropatterned PDMS substrate with linear grooves, PDMS (base:catalyst = 10:1) was poured onto the mold and cured at 70 °C for 2 h. For the control experiments, flat, unpatterned PDMS substrates were made by pouring the PDMS (base:catalyst = 10:1) into a plastic dish and cured under the same conditions.

### 2.2. Cell Culture on the Micropatterned PDMS Substrate

The micropatterned PDMS substrate, approximately 1-mm thick, was attached to a 35-mm dish (Figure S1A) and irradiated with UV light for sterilization. Subsequently, the surfaces of the PDMS substrates were modified with 3-Glycidyloxypropyltrimethoxysilane (Sigma-Aldrich, St. Louis, MO, USA), and mouse laminin (Corning Inc., Corning, NY, USA) diluted in PBS at 30 µg/mL was placed on the substrates at 3 μg/cm^2^. After the laminin solution was dried on a clean bench, the substrate was rinsed twice with PBS.

C2C12 cells, a mouse SkM myoblast cell line, were cultured and passaged as previously described [12]. C2C12 cells were seeded on the coated PDMS substrates at a density of 1 × 10^4^ cells/cm^2^ in DMEM supplemented with 10% FBS and 1% PS. After 3 days, the medium was changed to differentiation medium (DMEM supplemented with 2% horse serum, 1% PS, and 1% Matrigel). The differentiation medium was changed every other day. Hu5/KD3 [13], immortalized human SkM cells, were provided by Dr. Naohiro Hashimoto and cultured and passaged as reported previously [14]. Hu5/KD3 cells were seeded on coated PDMS substrates at a density of 2 × 10^4^ cells/cm^2^ in DMEM supplemented with 20% FBS, 0.5% PS, 2 mM L-glutamine, and 2% Ultroser G serum substitute (Sartorius, Göttingen, Germany). After 3 days, the medium was replaced with the differentiation medium. The differentiation medium was changed every other day.

### 2.3. Fluorescent Staining of Cells Cultured on the PDMS Substrates

After 5 days of cultivation in differentiation medium, the cells were fixed with 4% paraformaldehyde (163-20145, Wako, Osaka, Japan) for 20 min, washed three times with PBS, and permeabilized with 0.3% Triton X-100/PBS (T8787, Sigma-Aldrich, St. Louis, MO, USA) for 5 min. Subsequently, the cells were washed three times with PBS for 10 min each and blocked with a blocking solution (PBS containing 10% goat serum and 0.01% TritonX-100) for 1 h at 25 °C. The cells were incubated with the primary antibody (monoclonal anti-α-actinin, A7811, Sigma-Aldrich, St. Louis, MO, USA) overnight at 4 °C. The next day, the cells were washed with PBS three times for 10 min each and incubated with the secondary antibody (CF555 goat anti-mouse IgG (H + L), 20231, Biotium, Fremont, CA, USA), Alexa Fluor™ 488 conjugated α-bungarotoxin (α-BTX, Invitrogen, Gaithersburg, MD, USA), and 4′,6-diamidino-2-phenylindole (DAPI) for 1 h at room temperature. Cells were observed under a BZ-X710 microscope (KEYENCE, Osaka, Japan).

The fluorescence images were analyzed. The orientation angles of the myotubes were quantified using fiber orientation analysis software version 8 (http://www.enomae.com/FiberOri/index.htm, accessed on 23 November 2022) [15]. AChR clustering on myotubes was quantified using ImageJ software version 1.52 (https://imagej.nih.gov/ij/, accessed on 23 November 2022) by dividing the total area of the 5 μm^2^ ≥ AChR clusters labeled with α-BTX [16] by the total area of the α-actinin-positive area in the immunofluorescence images. In addition, using the ImageJ software, the number of nuclei in the α-actinin-positive area was quantified as the fusion index, and the myotube width was quantified by measuring the width of the α-actinin-positive myotubes in the immunofluorescence images.

### 2.4. Real-Time PCR

Total RNA was extracted from cells cultured on PDMS substrates using Nucleospin RNA (Macherey-Nagel Inc., Düren, Germany), according to the manufacturer’s instructions. An amount of cDNA was prepared from the extracted RNA using ReverTra Ace qPCR RT Master Mix with gDNA Remover (Toyobo, Osaka, Japan), according to the manufacturer’s instructions. The cDNA was quantified by using Qubit Fluorometer (Thermo Fisher Scientific, Waltham, MA, USA). Real-time PCR was performed using the StepOne Real-Time PCR system (Applied Biosystems, Waltham, MA, USA) with the THUNDERBIRD SYBR qPCR Mix (Toyobo, Osaka, Japan). The PCR primers were purchased from FASMAC (Kanagawa, Japan). The primer sequences used are listed in Table 1.

### 2.5. Co-Culture of Human SkM Myotube and Human iPSC-Derived MNs

Human iPSC clones (201B7 [17]) provided by Dr. Shinya Yamanaka (RIKEN BRC through the Project for Realization of Regenerative Medicine and National Bio-Resource Project of the MEXT, Japan) were cultured and differentiated into MNs, as reported previously [18,19]. Some hiPSC-derived MN spheroids cultured for 14 or 15 days were collected in a 15 mL tube and washed twice with PBS. After the removal of PBS, 500 µL of a 1:1 mixture of TrypLE and EDTA (0.5 mM) was added to the tube and incubated at 37 °C for 4 min. Next, 500 µL of 1 mg/mL trypsin inhibitor (Roche, Basel, Switzerland) was added and the solution was pipetted to dissociate the spheroid. The solution was passed through a 40 µm cell strainer (Corning Inc., Corning, NY, USA) to obtain a single-cell suspension of iPSC-derived MNs.

Hu5/KD3 cells were cultured in differentiation medium on a PDMS substrate. On day 2, the medium was aspirated, and iPSC-derived MNs suspended in the MN medium [20] were seeded onto the PDMS substrate at a density of 5 × 10^4^ cells/cm^2^. After 24 h, the medium was aspirated, and KBM neural stem cell (Kohjin Bio, Saitama, Japan) supplemented with 25% Matrigel were added. Subsequently, the dish was incubated at 37 °C for 30 min, resulting in cells on the PDMS substrate being covered with a thin gel layer of Matrigel. For the subsequent co-culture, a 1:1 mixture of Hu5/KD3 differentiation medium and MN medium was used. The medium was changed every two days.

Fluorescent staining was performed as described in Section 2.3. Mouse MF 20 (Developmental Studies Hybridoma Bank (DSHB), Iowa City, IA, USA) and rabbit anti-β tubulin (802001, BioLegend, San Diego, CA, USA) were used as the primary antibodies. Alexa Fluor 350 goat anti-mouse IgG1 (Invitrogen, Waltham, MA, USA) and CF543 goat anti-rabbit IgG (H + L) (20330, Biotium, Fremont, CA, USA) were used as the secondary antibodies. Alexa Fluor™ 488 conjugated α-bungarotoxin (Invitrogen, Waltham, MA, USA) was used to visualize the AChR clustering. Cells were observed under a BZ-X710 microscope (KEYENCE, Osaka, Japan).

### 2.6. Assessment of Functionality of NMJs

The presence of NMJs in co-cultured Hu5/KD3 cells and iPSC-derived MNs was examined by observing the muscle contraction induced by the addition of neurotransmitters via NMJ. The cells were co-cultured for five days on the PDMS substrate. Four hundred microliters of L-glutamate solution (Glu), a neurotransmitter, was added to the dish with co-cultured cells to induce MN firing. Thereafter, 2 µM of tetrodotoxin (TTX, Wako, Osaka, Japan) was added to the dish with co-cultured cells to inhibit MN firing. Contraction of Hu5/KD3 myotubes, which was induced and inhibited by the addition of Glu and TTX, was observed under an inverted phase-contrast microscope (CKX53, Olympus, Tokyo, Japan) equipped with a camera (DP21, Olympus, Tokyo, Japan). Motion tracking software (PV Studio 2D, OA Science, Miyazaki, Japan) was used to analyze contractile myotube movement.

### 2.7. Statistical Analysis

The data were presented as mean ± standard deviation (SD). Two-tailed unpaired *t*-tests were used to assess differences between two groups with equal variance using Prism 7 (GraphPad Software, La Jolla, CA, USA).

## 3. Results and Discussion

### 3.1. Alignment and AChR Clustering of SkM Cells Cultured on the Surface with Linear Grooves

**Figure 1:**
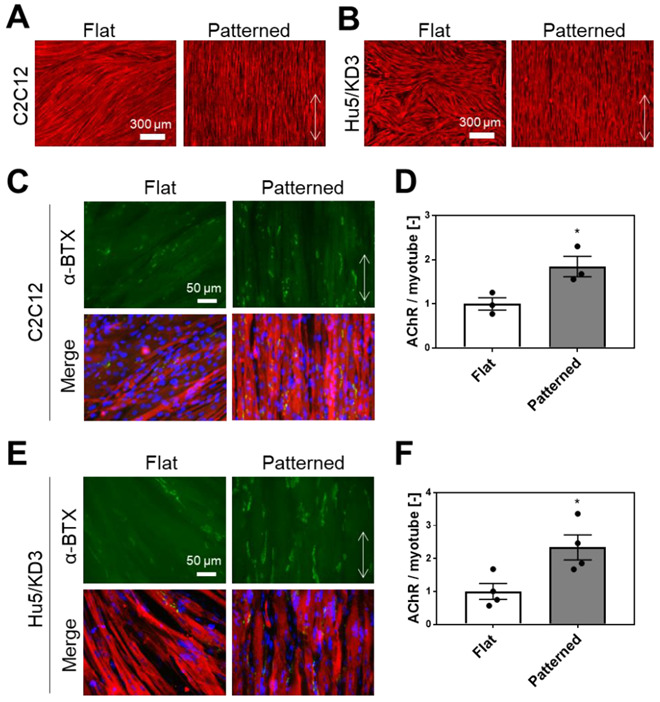
Alignment and AChR clustering of SkM cells cultured on the surface with linear grooves. Immunofluorescence staining of (**A**) C2C12 and (**B**) Hu5/KD3 myotube cultured on the flat or patterned surface. Red indicates α-actinin. Scale bar: 300 µm. Fluorescence staining of AChR clustering in (**C**) C2C12 and (**E**) Hu5/KD3 myotube cultured on the flat or patterned surface. Green indicates AChR clustering, red indicates α-actinin, and blue indicates nuclei. Scale bar: 50 µm. Number of AChR clustering in (**D**) C2C12 or (**F**) Hu5/KD3 myotubes. Data points represent means ± SD (*n* = 3–4). Double arrow indicates the direction of linear grooves. * *p* < 0.05.

We investigated whether the micropatterned PDMS substrate with linear grooves (Figure S1A) enhanced AChR clustering in myotubes cultured on their surfaces. Mouse C2C12 myoblasts and human Hu5/KD3 myoblasts were used in this study. C2C12 and Hu5/KD3 myoblasts were cultured and differentiated into myotubes on surfaces with grooves. The myotubes were aligned along the direction of the linear grooves (Figure 1A,B). Because the orientation angles of the myotubes were similar for all sizes (5, 10, 15, and 20 µm) of the lines and spaces on the PDMS substrate (Figure S1B,C), we did not segregate the sizes of the pattern and randomly observed myotubes on the substrate in subsequent experiments (Figure S1A).

The formation of AChR clusters was visualized using fluorescently labeled α-BTX. It was observed that the AChR clustering in myotubes of both C2C12 and Hu5/KD3 was facilitated by culturing on the patterned surface (Figure 1C,E). Quantitative analysis indicated that the number of AChR clusters per myotube area increased 1.85 times for C2C12 (Figure 1D) and 2.34 times for Hu5/KD3 compared to the flat surface (Figure 1F).

Ko et al., reported an increase in AChR clustering in mice-derived SkM cells, primary myoblasts, and C2C12 myoblasts cultured on surfaces with linear grooves coated with Matrigel [10]. In this study, we showed that AChR clustering was enhanced in both mouse- and human-derived SkM cells cultured on a grooved PDMS surface coated with laminin (Figure 1). Considering this result, although further experiments using various SkM cells including human primary cells are needed, the increase in AChR clustering induced by patterned surfaces may be widely observed for SkM cells regardless of their origin.

### 3.2. Expression Analysis of Key AChR Clustering Genes in SkM Cells Cultured on a Surface with Linear Grooves

To examine the possible mechanism of AChR clustering enhancement observed by culturing cells on a linearly patterned surface (Figure 1), we cultured C2C12 myoblasts on the patterned surface and performed gene expression analysis.

**Figure 2:**
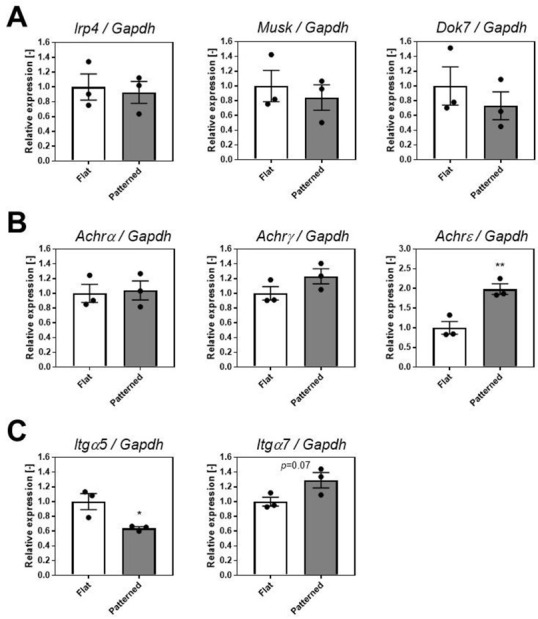
Gene expression analysis of C2C12 cells cultured on the patterned surface. (**A**) *Lrp4*, *Musk*, and *Dok7*. (**B**) *Achrα*, *Achrγ*, and *Achrε*. (**C**) *Itgα5* and *Itgα7*. Data are expressed relative to *Gapdh*. Data points represent mean ± SD (*n* = 3). * *p* < 0.05 versus flat. ** *p* < 0.01 versus flat.

First, we examined the gene expression of low-density lipoprotein receptor-related protein 4 (Lrp4), muscle-specific receptor tyrosine kinase (Musk), and docking protein 7 (Dok7), which play key roles in the process of in vivo AChR clustering and NMJ formation. Motor neuron-derived Agrin binds to Lrp4 on the muscle cell membrane, further stimulating the interaction between Lrp4 and MuSK, and increases MuSK phosphorylation [21]. Dok7 stabilizes and promotes MuSK tyrosine phosphorylation, and downstream signaling from MuSK leads to AChR clustering and NMJ formation [21]. As shown in Figure 2A, the expression levels of *Lrp4*, *Musk*, and *Dok7* did not differ between the flat and patterned groups.

Next, we examined the gene expression of the AChR subunits. AChR forms a heteropentamer consisting of two α-, one β-, and one δ-subunits with one γ- or ε-subunit for fetal or adult, respectively [22]. We measured the gene expression of α-, γ-, and ε-subunits. As shown in Figure 2B, the gene expression of *Achrα* and *Achrγ* was similar for the flat and patterned substrates, whereas the expression of *Achrε* increased significantly for the patterned substrates. This result suggests that the number of adult α2βδε AChR clusters increases upon culturing on a linearly patterned surface. AChR interacts with the cytoskeleton directly or indirectly via proteins, such as rapsyn [23]. Overexpression of rapsyn in quail fibroblasts transfected with AChR subunits induces AChR clustering [24]. Thus, it would be interesting to clarify the involvement of proteins that bind to AChR, including rapsyn, in the increase in AChR clusters on a linearly patterned surface in the future.

We also examined the gene expression of integrins α5 (*Itgα5*) and α7 (*Itgα7*). The α5β1 integrin is a classical fibronectin receptor and α7β1 integrin is a laminin receptor in SkM. The α5β1 integrin is downregulated after myotube formation, whereas α7β1 integrin is upregulated upon myoblast fusion [25]. It was previously reported that adding an antibody against α7β1 integrin to rat myofibers inhibited laminin-induced AChR clustering [26]. As shown in Figure 2C, gene expression of *Itgα7* increased, whereas that of *Itgα5* decreased when patterned. This result suggests that culturing on a linearly patterned surface increases the number of α7β1 integrins in the myotubes. Because we coated the culture surface with laminin, it is possible that the increase in the number of α7β1 integrins by culturing on the patterned surface enhanced the laminin-induced AChR clustering pathway [27,28].

### 3.3. Effects of Patterned Surface on the Differentiation of SkM Cells

**Figure 3:**
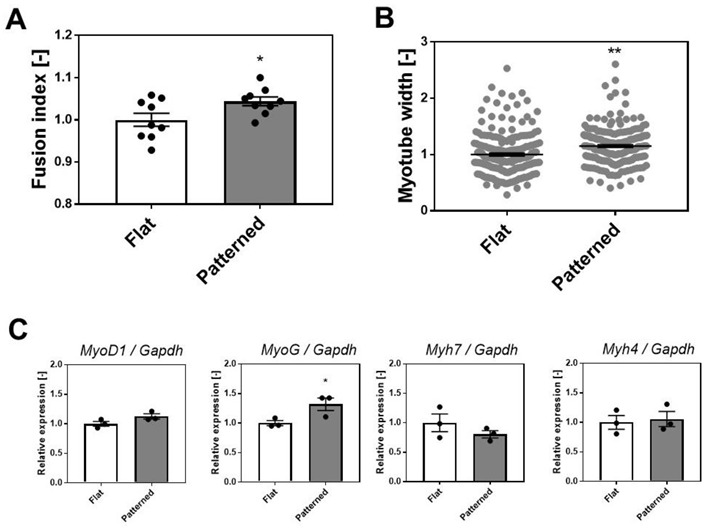
Degree of differentiation of C2C12 cells cultured on the patterned surface. (**A**) Fusion index. Data points represent mean ± SD, quantified from nine observations. (**B**) Myotube width. Data points represent mean ± SD, quantified from myotubes (215 for flat and 216 for patterned) in nine observations. (**C**) Gene expression of representative marker genes for SkM development (*MyoD1*, *MyoG*, *Myh7*, and *Myh4*). Data points represent mean ± SD (*n* = 3). * *p* < 0.05 versus flat. ** *p* < 0.01 versus flat.

As shown in Figure 2B,C, the expression of *the Achrε* and *Itgα7* genes increased in contrast to the decrease in expression of the *Itgα5* gene. These results suggest that myotu-bes cultured on patterned surfaces with linear grooves are better differentiated than those cultured on flat surfaces. Therefore, to examine the degree of differentiation, we compared the number of nuclei within myotubes (fusion index) and the myotube width cultured on each surface. C2C12 myotubes and nuclei on day 5 of differentiation were fluorescently visualized with α-actinin and DAPI, respectively, and the number of nuclei in the α-actinin-positive area was quantified as a fusion index by image analysis. The fusion index of the patterned surface was significantly higher than that of the flat surface (Figure 3A). In addition, the width of myotubes on the patterned surface was significantly larger than that on the flat surface (Figure 3B).

We also examined the expression of representative marker genes for SkM development, including myoblast determination protein 1 (*MyoD1*), myogenin (*MyoG)*, and myosin heavy chain (Myh) isoforms (*Myh7* and *Myh4*). *MyoD1* and *MyoG* are muscle-specific transcription factors. *Myh7* and *Myh4* are slow or fast Myh isoforms that are considered primary or secondary myogenesis markers, respectively [29]. Although the expression of *MyoD1*, *Myh7*, and *Myh4* did not change, the expression of *MyoG* increased in SkM cells cultured on the patterned surface (Figure 3C). Because *MyoG* controls the terminal differentiation of myoblasts fusing with each other and forming multinucleated myotubes [29], this result may partly explain the increase in the fusion index (Figure 3A). Although further experiments of gene expression analysis for other differentiation markers, including dystrophin, are needed to understand the effects of patterned surfaces on the differentiation of SkM cells in detail, these results suggest that the differentiation of SkM cells was enhanced by culturing on PDMS with a linearly patterned surface.

Several studies have also reported that culturing SkM myotubes on a linear patterned surface enhanced the differentiation of myotubes, such as sarcomere formation, contractile protein content, and Ca^2+^ response to electric stimulation, compared to culturing on an unpatterned surface [30,31]. Similar to these earlier studies, the grooved PDMS surface coated with laminin used in this study enhanced the myotube differentiation. Thus, it is suggested that better-differentiated SkM myotubes increase the expression of α7β1 integrin and adult AChR, increasing AChR clustering.

### 3.4. Co-Culture of Human SkM Cells and Human iPS-Derived Motor Neurons on a Surface with Linear Grooves

Finally, to examine the usefulness of patterned surfaces for in vitro human NMJ models, we co-cultured human SkM cells and human iPSC-derived motor neurons on micropatterned PDMS with linear grooves and investigated whether the formation of NMJ was increased by culturing on the patterned surface.

**Figure 4:**
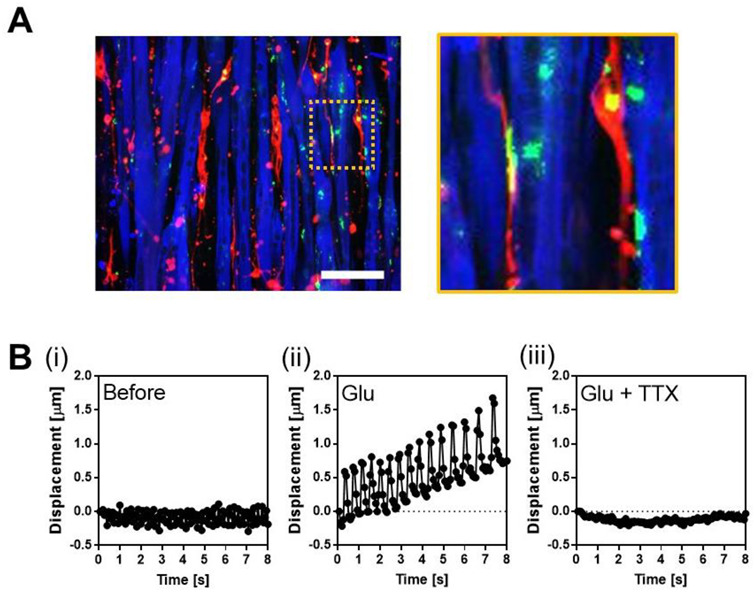
Co-culture of human SkM cells and MNs. (**A**) Immunofluorescent staining of co-culture on the linearly patterned surface. Green indicates AChR clustering, red indicates axon, and blue indicates MyHC. NMJs are surrounded by a dashed square, and a magnified view of this region is shown. Scale bar: 100 µm. (**B**) Representative contractile displacement of Hu5/KD3 myotubes co-cultured with iPSC-derived MNs on the patterned surface. (**i**) Before. (**ii**) After addition of glutamate (Glu). (**iii**) After addition of Glu and tetrodotoxin (TTX).

Glutamate (Glu) was used to induce MN firing and tetrodotoxin (TTX) was used to inhibit MN firing. We determined whether the myotubes co-cultured with MNs had NMJs based on the responses to the addition of these chemicals; they contracted by the addition of Glu, and the contraction was stopped by the addition of TTX. We prepared 12 co-cultured samples for patterning and flattening as controls and assessed the functionality of NMJs. Consequently, although myotubes with NMJs were observed for both patterned and flat (Figure 4 and Video S1), the number of samples with NMJs was higher for patterned than for flat (Table 2).

The number of samples with NMJs increased 2.5 times for patterns compared to flat (Table 2). This suggests that a patterned surface can be used for the effective screening of drugs that alter NMJs’ function of NMJs. However, the efficiency of NMJ formation was still low; we did not observe NMJs in 7 out of the 12 samples. As we assessed the functionality of NMJs on day 5 of co-culture (Figure 4), a longer co-culture duration may increase the efficiency of NMJ formation. In addition, an increase in the number of seeded MNs enhances NMJ formation.

Although a co-culture of mice-derived SkM cells and neurons on patterned surfaces has been reported [10], to the best of our knowledge, no studies have reported the co-culture of human-derived SkM cells and MNs on patterned surfaces. This is the first report to demonstrate that a patterned surface with linear grooves is useful for the effective formation of NMJs derived from human cells. However, in this study, we demonstrated co-culture using immortalized human skeletal muscle cells Hu5/KD3 [13]. In future studies, we will investigate whether the formation of NMJs between human iPSC-derived SkM cells and MNs is accelerated by the patterned surface to establish a more practical in vitro disease model.

Furthermore, it is possible that a patterned surface with linear grooves can be applied to the mixed co-culture models used in this study and other in vitro NMJ models [32,33]. Badu-Mensah et al., developed compartmentalized microfluidic devices for co-culture of SkM cells and MNs [32]. The devices had two chambers, one for SkM cells and the other for MNs, and the chambers were connected by micro-tunnels for MN axons. Considering the results of this study (Figure 3), it is expected that using a patterned surface with linear grooves for SkM cell chamber in compartmentalized microfluidic devices will enhance the differentiation of SkM cells. Although further experiments are needed to examine the effects of flat and patterned surfaces on the activity of MNs, the application of a linearly patterned surface to the device may increase NMJ formation.

## 4. Conclusions

In this study, we examined the effects of the patterned surface on AChR clustering in myotubes and found that better differentiation of myotubes on the patterned surface induced the gene expression of integrin α7 and AChR ε-subunit, thereby increasing AChR clustering. We also found that the number of NMJs between human SkM cells and MNs increased upon co-culture on a linearly patterned surface. Thus, it was suggested that a patterned surface would be useful for creating in vitro human NMJ models.

## Figures and Tables

**Table 1:** Primers used in this study.

Gene	Orientation	Sequence (5′-3′)
*Gapdh*	Forward	TCAACAGCAACTCCCACTCTTCCA
	Reverse	ACCACCCTGTTGCTGTACCGTATT
*Lrp4*	Forward	GGAGGGTCAGTGAAGATGTAAAG
	Reverse	CTGGCTGCTGATCTCTGAATAG
*Musk*	Forward	CATGGCAGAGTTTGACAACCC
	Reverse	TTCGGAGGAACTCATTGAGGTC
*Dok7*	Forward	TGAGCTTCCTGTTTGACTGCA
	Reverse	GCAACACGCTCTTCTGAGGC
*Itgα5*	Forward	ACCTGGACCAAGACGGCTACAA
	Reverse	CTGGGAAGGTTTAGTGCTCAGTC
*Itgα7*	Forward	AACCAATGGCTGGGAGTCAG
	Reverse	ATCCCGAGTCTCCAAAGCCT
*Achrα*	Forward	ACCTGGACCTATGACGGCTCT
	Reverse	AGTTACTCAGGTCGGGCTGGT
*Achrγ*	Forward	CTTGTGGCTAAGAAGGTGCCTG
	Reverse	GCAAGGACACATTGAGCACGAC
*Achrε*	Forward	AGACCTGAGGACACTGTCACCA
	Reverse	TCGTCCTTGCTGTAGTTGAGCC
*MyoD*	Forward	AGGACACGACTGCTTTCTTCACCA
	Reverse	TTAACTTTCTGCCACTCCGGAACC
*MyoG*	Forward	CCAACCCAGGAGATCATTTG
	Reverse	ACGATGGACGTAAGGGAGTG
*Myh7*	Forward	ATGCTGACAGATCGGGAGAA
	Reverse	GGTTGGCTTGGATGATTTGA
*Myh4*	Forward	GGCACCCTTGAGGATCAAAT
	Reverse	GCTATCAATGTCCGCAGAGG

**Table 2:** Comparison of the number of functional NMJ observed in co-culture models of human SkM myotubes and human iPSC-derived MNs.

	Total Number of Observations [-]	Number of Observations Including Myotubes Contracted by Glutamate Addition [-]
Flat	12	2
Patterned	12	5

## Data Availability

Not applicable.

## Supplementary Materials

The following supporting information can be downloaded at: https://www.mdpi.com/article/10.3390/cells11233760/s1, Figure S1: Alignment of myotubes cultured on the patterned surface. Video S1: Representative contractile displacement of Hu5/KD3 myotubes co-cultured with iPSC-derived MNs on the patterned surface.
