# Supplementary material for: Alignment of Skeletal Muscle Cells Facilitates Acetylcholine Receptor Clustering and Neuromuscular Junction Formation with Co-Cultured Human iPSC-Derived Motor Neurons
Source: Cells. 2022 Nov 24;11(23):3760. doi: 10.3390/cells11233760 (PMC9738074; doi:10.3390/cells11233760)
Supplement: Supplementary file 1 [file cells-11-03760-s001.zip › cells-1956316 supplementary Proof back/cells-1956316 supplementary proof back.pdf]

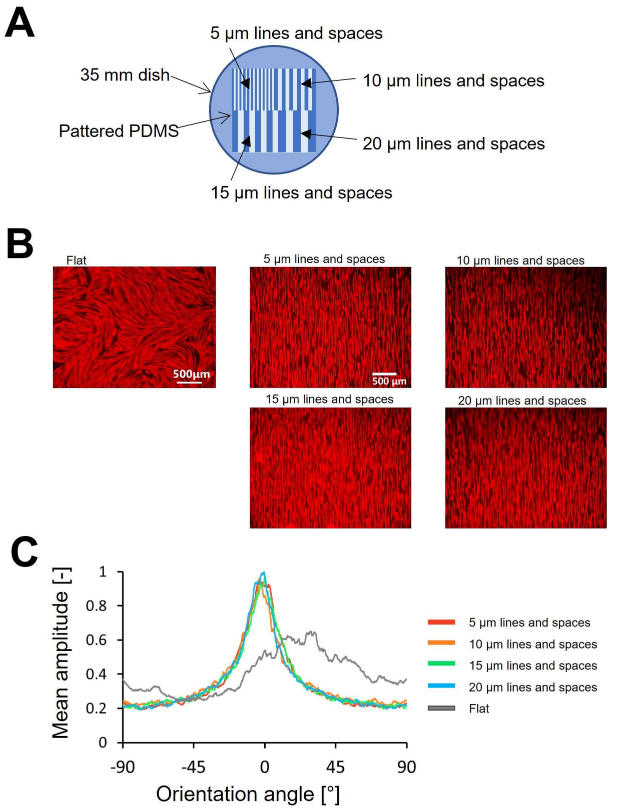

Figure S1: Alignment of myotubes cultured on the patterned surface. A) The micropatterned PDMS substrate used in this study.B) Immunofluorescence staining of C2C12 myotubes cultured on the flat or patterned surface (5,10,15, and 20 μm lines and spaces) C) Orientation angle of C2C12 myotubes cultured on the flat or patterned surface (5,10,15, and 20 μm lines and spaces).
